# Comparison of image quality and spatial resolution between ^18^F, ^68^Ga, and ^64^Cu phantom measurements using a digital Biograph Vision PET/CT

**DOI:** 10.1186/s40658-022-00487-7

**Published:** 2022-09-05

**Authors:** Anja Braune, Liane Oehme, Robert Freudenberg, Frank Hofheinz, Jörg van den Hoff, Jörg Kotzerke, Sebastian Hoberück

**Affiliations:** 1 Department of Nuclear Medicine, University Hospital Carl Gustav Carus at the Technische Universität Dresden, Dresden, Germany; 2 PET Center, Institute of Radiopharmaceutical Cancer Research, Helmholtz-Zentrum Dresden-Rossendorf, Dresden, Germany; 3 Department of Nuclear Medicine, Medizinische Fakultat Carl Gustav Carus, University Hospital Carl Gustav Carus at the Technische Universität Dresden, Dresden, Germany; 4 Department of Nuclear Medicine, Helios Klinikum Erfurt, Erfurt, Germany

**Keywords:** PET/CT, ^18^F-FDG, ^68^Ga, ^64^Cu, NEMA PET body phantom, Jaszczak phantom, Spatial resolution, Image quality

## Abstract

**Background:**

PET nuclides can have a considerable influence on the spatial resolution and image quality of PET/CT scans, which can influence diagnostics in oncology, for example. The individual impact of the positron energy of ^18^F, ^68^Ga, and ^64^Cu on spatial resolution and image quality was compared for PET/CT scans acquired using a clinical, digital scanner.

**Methods:**

A Jaszczak phantom and a NEMA PET body phantom were filled with ^18^F-FDG, ^68^Ga-HCl, or ^64^Cu-HCl, and PET/CT scans were performed on a Siemens Biograph Vision. Acquired images were analyzed regarding spatial resolution and image quality (recovery coefficients (RC), coefficient of variation within the background, contrast recovery coefficient (CRC), contrast–noise ratio (CNR), and relative count error in the lung insert). Data were compared between scans with different nuclides.

**Results:**

We found that image quality was comparable between ^18^F-FDG and ^64^Cu-HCl PET/CT measurements featuring similar maximal endpoint energies of the positrons. In comparison, RC, CRC, and CNR were degraded in ^68^Ga-HCl data despite similar count rates. In particular, the two smallest spheres of 10 mm and 13 mm diameter revealed lower RC, CRC, and CNR values. The spatial resolution was similar between ^18^F-FDG and ^64^Cu-HCl but up to 18% and 23% worse compared with PET/CT images of the NEMA PET body phantom filled with ^68^Ga-HCl.

**Conclusions:**

The positron energy of the PET nuclide influences the spatial resolution and image quality of a digital PET/CT scan. The image quality and spatial resolution of ^68^Ga-HCl PET/CT images were worse than those of ^18^F-FDG or ^64^Cu-HCl despite similar count rates.

**Supplementary Information:**

The online version contains supplementary material available at 10.1186/s40658-022-00487-7.

## Background

Currently, different radiotracers are available for the examination of the same disease, such as ^18^F-PSMA-1007 and ^68^Ga-PSMA-11 for the diagnosis of prostate cancer [1–3] or ^68^Ga-DOTA-TOC and ^18^F-SiFAlin-TATE for the diagnosis of neuroendocrine tumors [4]. In a clinical study examining 102 patients, in repeated PET measurements of the same patient using the same PET/CT scanner, ^18^F-PSMA-1007-PET revealed approximately five times as many PSMA-positive lesions attributed to benign origin as ^68^Ga-PSMA-11-PET [1, 2]. The same study revealed that the maximum standardized uptake value (SUV_max_) of lesions attributed to benign origin was higher for ^18^F-PSMA-1007-PET than for ^68^Ga-PSMA-11-PET [2]. These differences between PET scans of different radionuclides might be caused by a combination of different effects: Radiotracers differ in their biokinetics and affinities [5]. In addition, state-of-the-art digital PET/CT scanners allow PET measurements with higher sensitivity and spatial resolution. This could reveal differences in PET scans of different nuclides that were previously undetectable. The more precise measurements also offer new possibilities for analyses of the impact of different radionuclides and their physical properties on image quality and detectability of small lesions. The maximum positron energy and thus the mean range in tissue differ between ^68^Ga, ^18^F, and ^64^Cu: Positrons arising from the decay of ^68^Ga feature an endpoint energy three times higher than those of ^18^F or ^64^Cu and therefore have a much greater mean range in tissue (^68^Ga: 3.5 mm; ^18^F: 0.6 mm; ^64^Cu: 0.7 mm) (Table 1) [6]. This degrades the spatial resolution of ^68^Ga-PET.

**Table 1:** Physical properties of the positron-emitting radioisotopes ^18^F, ^68^Ga, and ^64^Cu [16]

	^18^F	^68^Ga	^64^Cu
β^+^ yield (%)	96.7	88.0 (1899 keV)	17.4
		1.1 (822 keV)	
Half-life (min)	109.8	67.6	762
Maximum endpoint β^+^ energy (keV)	633.5	1899.1	653.1
Average β^+^ energy (keV)	249.3	836.0	278
Maximum range of positrons in water (mm)	2.4	9.2	2.5
Mean range of positrons in water (mm)	0.6	3.5	0.7

The individual impact of different physical properties of PET nuclides on PET image quality and spatial resolution has already been studied in detail, including the use of preclinical PET/CT scanners with a higher intrinsic spatial resolution than clinical PET/CT scanners [7–10]. In addition, different simulations have been performed on this topic [6, 10–12]. However, no analyses have been performed on PET data recorded on PET/CT scanners of the newest generation, which allow for more accurate PET measurements by applying time-of-flight (ToF) measurements and point spread function (PSF) reconstruction. Such devices might reveal new details on the influence of different positron energies of ^68^Ga, ^18^F, or ^64^Cu on image quality and detectability of small lesions.

Therefore, the aim of this work was to compare the individual impact of the positron energy of the most commonly used PET isotopes ^18^F and ^68^Ga as well as ^64^Cu on the image quality and spatial resolution of scans acquired with one of the latest clinical digital PET/CT scanners. This study focused specifically on the influence of the positron energy of the PET nuclide on image quality. All other factors that could influence PET image quality in addition to the positron energy, such as count rate, PET device, phantom setup, and reconstruction parameters, were kept in a comparable range. The scans were recorded with a Biograph Vision PET/CT scanner (Siemens Healthineers) enabling ToF measurements with a timing resolution of 214 ps and an axial spatial resolution of 3.7 mm full width at half maximum (FWHM) for a point source positioned 10 cm from the center of the plane [13]. Due to the very good imaging properties of the scanner, deterioration is to be expected in the ^68^Ga images due to the increased positron range.

## Methods

**Fig. 1:**
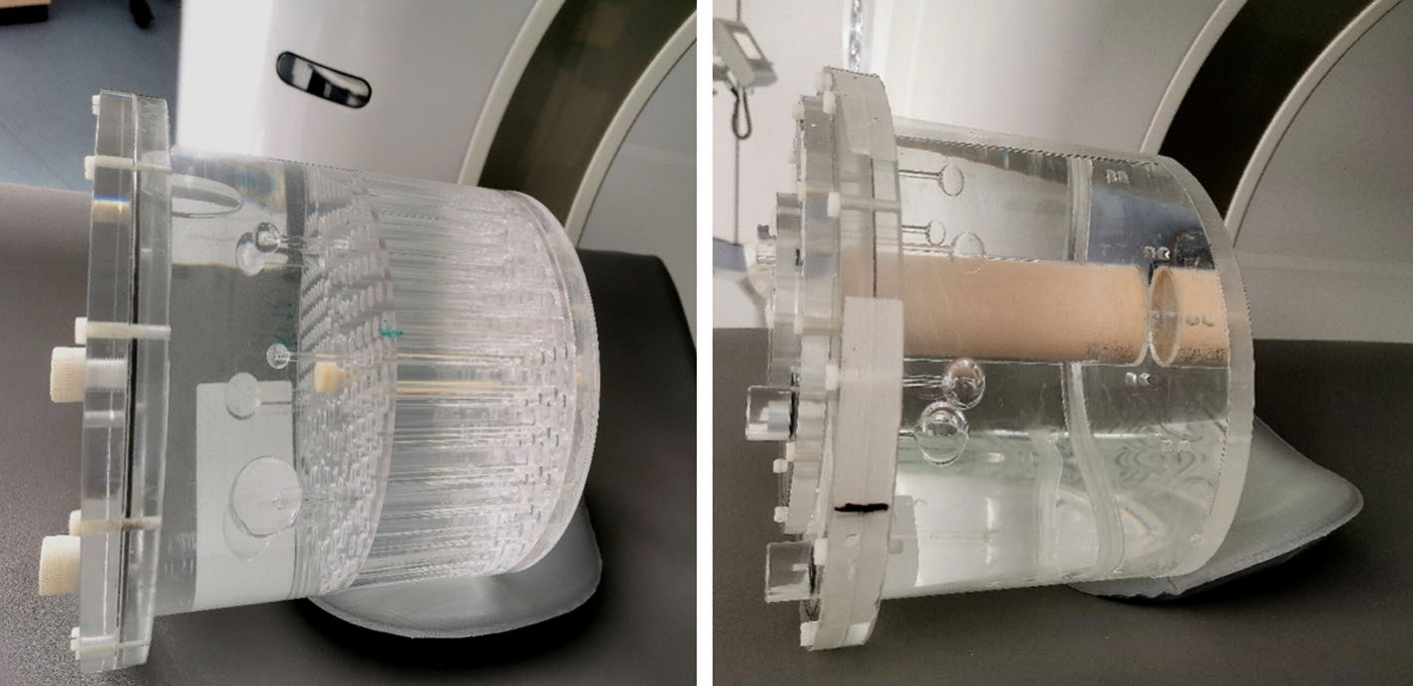
Jaszczak phantom (left) and NEMA PET body phantom (right). The Jaszczak phantom was used for qualitative evaluation of spatial resolution and image quality. It features cold rods (4.8 mm, 6.4 mm, 7.9 mm, 9.5 mm, 11.1 mm, and 12.7 mm in diameter) and spheres (9.5 mm, 12.7 mm, 15.9 mm, 19.1 mm, 25.4 mm, and 31.8 mm in diameter) surrounded by an activity-filled background compartment. The NEMA PET body phantom (right) was used for semiquantitative assessment of image quality. It features spheres of different sizes (10 mm, 13 mm, 17 mm, 22 mm, 28 mm, and 37 mm in diameter), which can be filled with radioactivity allowing hot spot imaging. The spheres are surrounded by a background compartment, which can also be filled with radioactivity. The cylindrical lung insert is positioned in the center of the phantom. It simulates patient lung tissue and features a density similar to lung tissue

A Jaszczak phantom (Model ECT/DLX/P, Data Spectrum Corporation, Durham, USA) was used to qualitatively evaluate spatial resolution and image quality. It is illustrated in Fig. 1. The Jaszczak phantom features cold rods (4.8 mm, 6.4 mm, 7.9 mm, 9.5 mm, 11.1 mm, and 12.7 mm in diameter) and spheres (9.5 mm, 12.7 mm, 15.9 mm, 19.1 mm, 25.4 mm, and 31.8 mm in diameter) surrounded by an activity-filled background compartment (0.3 mol hydrochloric acid (HCl)). PET measurements of the Jaszczak phantom are common for the analysis of spatial resolution and enable a visual assessment of the separability of the cold rods. However, focal hot spots cannot be imitated as in real patient data with the Jaszczak phantom. We additionally determined the spatial resolution semiquantitatively using the NEMA PET body phantom (PTW Freiburg), as shown in Fig. 1. The NEMA PET body phantom allows hot spot imaging of spheres of different sizes (10 mm, 13 mm, 17 mm, 22 mm, 28 mm, and 37 mm in diameter) at different sphere-to-background contrast ratios.

All PET measurements were performed on a digital Biograph Vision PET/CT system (Siemens Healthineers). Following a low-dose CT (120 kVp, 78 mAs, spiral pitch factor of 1.5, 512 × 512 matrix with a pixel size of 0.98 mm × 0.98 mm) used for attenuation correction of the subsequent PET scan, the PET data were acquired in the list mode over a single bed position, covering an axial field of view (FoV) of 26 cm [13]. The duration of the PET data acquisition of the Jaszczak phantom filled with ^18^F-FDG was determined based on the recommendations in the NEMA NU 2-2018 protocol for the characterization of image quality [14]. In the corresponding measurements with the NEMA PET body phantom, the standards in clinical routine according to the German Guideline for ^18^F-FDG PET/CT in oncology were used as a reference [15]. The duration of the acquisition of the ^68^Ga-HCl and ^64^Cu-HCl PET data was adjusted to that of the ^18^F-FDG-PET data. The respective actual activity concentration in the phantom at the timepoint of imaging and the decay probability of the nuclides (Table 1) were taken into account to achieve similar count statistics between scans with different nuclides. PET data were reconstructed according to the standards in our clinical routine for ^18^F-FDG using an ordered subset expectation maximization (OSEM) 3D iterative reconstruction algorithm with 6 iterations and 5 subsets (6i5s), applying PSF and ToF (TrueX algorithm) with an image matrix size of 440 × 440, resulting in a voxel size of (1.65 × 1.65 × 1.5) mm^3^. No postfiltering was applied (all-pass filter). Reconstructions were performed with attenuation correction and relative scatter correction.

### Qualitative evaluation of spatial resolution using the Jaszczak phantom

The background compartment of the Jaszczak phantom was filled with ^18^F-FDG, ^68^Ga-HCl, or ^64^Cu-HCl aiming at an activity concentration of 5.3 kBq/mL [14].

The acquisition duration of the Jaszczak phantom filled with ^18^F-FDG, ^68^Ga-HCl, or ^64^Cu-HCl was 546 s, 611 s, or 2629 s, respectively. PET/CT scans of the Jaszczak phantom were analyzed visually to determine spatial resolution. In each PET/CT scan, the narrowest rods and spheres that were still distinguishable from one another were determined visually.

### Semiquantitative evaluation of spatial resolution and image quality using the NEMA PET body phantom

The NEMA PET body phantom was filled with ^18^F-FDG, ^64^Cu-HCl, or ^68^Ga-HCl. According to the recommendations in the NEMA NU 2–2018 protocol, we aimed at an activity concentration of 5.3 kBq/ml in the background compartment and a sphere-to-background activity concentration ratio of approximately 4:1 or 8:1 [14]. The actual activity concentrations at the timepoint of imaging differed slightly from the target and are specified in Table 2. The phantom was also scanned after wrapping it in gel cooling packs 1 cm thick containing propylene glycol to simulate attenuation and scatter conditions comparable with those in an obese patient. The acquisition durations of all PET/CT scans of the NEMA PET body phantom are specified in Table 2.

**Table 2:** Information about measurements of the NEMA PET body phantom filled with ^18^F-FDG, ^68^Ga-HCl, or ^64^Cu-HCl

Nuclide	Weight setup	Sphere background activity concentration ratio	Activity concentration in spheres (kBq/ml)	Activity concentration in background compartment (kBq/ml)	Total number of true events (× 10^6^ counts)	Acquisition duration (s)
^18^F-FDG	Normal	4.0	18.21	4.51	70.8	357
	Obese	4.0	15.55	3.85	45.0	419
	Normal	9.7	51.97	5.35	72.4	291
	Obese	9.7	43.59	4.48	45.0	347
^68^Ga-HCl	Normal	4.3	18.75	4.40	71.3	395
	Obese	4.3	14.63	3.43	44.0	506
	Normal	9.4	40.65	4.32	69.9	393
	Obese	9.4	31.52	3.35	44.7	507
^64^Cu-HCl	Normal	5.4	27.70	5.12	72.2	1703
	Obese	5.4	26.18	4.84	45.0	1801
	Normal	8.1	42.16	5.19	71.6	1659
	Obese	8.1	40.04	4.93	44.6	1747

Activity concentration at the beginning of the acquisition and total number of detected true events throughout the duration of PET imaging

### Image analysis

Spatial resolution was evaluated semiquantitatively according to Hofheinz et al. [17] using the software Rover (version 3.0.60 h, ABX, Radeberg, Germany). Briefly, the resolution was determined based on the analysis of radial activity profiles of the homogeneously filled phantom spheres and the assessment of the FWHM of the PSF in the reconstructed images. PSF was modeled by a 3D Gaussian function, and FWHM was determined by applying the method described in detail in [17]. This method is based on fitting the analytic solution for the radial activity profile of a homogeneous sphere convolved with a 3D Gaussian function to the reconstructed data. In this process, the full 3D vicinity of each sphere is evaluated by transforming the data to spherical coordinates relative to the center of the sphere. The analytic solution has five parameters: signal (true activity within the sphere), background level, FWHM of the PSF, sphere radius, and wall thickness of the spherical inserts. The radius and wall thickness of the spheres were fixed to their known values. The remaining three parameters were determined by nonlinear least-squares fitting. With this method, the spatial resolution can be determined at a finite background as well as for extended objects. Therefore, it allowed us to study the size and contrast dependence of the resolution. Note that this method assumes a Gaussian PSF, which is never exactly the case. However, the method still leads to a reasonable approximation of the spatial resolution as long as the slope at the object boundary (signal decline) is modeled correctly by the fit function. The means and standard deviations of the FWHM of all six spheres were compared between PET measurements with different nuclides.

Image quality was evaluated semiquantitatively using Rover (version 3.0.60 h, ABX, Radeberg, Germany). Three uniform background volumes of interest (VOIs) of at least 61 ml volume were delimited as illustrated in Additional file 1: Fig. S1A. According to [18], a 3D isocontour at 50% of the maximum pixel value was used for the segmentation of each sphere, taking into account the activity concentration in the background of the phantom. For each sphere, the mean, maximum, and peak recovery coefficients (RC_mean_, RC_max_, and RC_peak_, respectively) were determined as the ratio of the measured mean, maximum, or peak SUV of the VOI to the actual activity concentration in the phantom sphere at the timepoint of imaging. SUV_peak_ was determined as the mean SUV of a 12-mm-diameter spherical region around the maximum voxel, considering only voxels within the 3D isocontour of the sphere [19]. The actual activity concentration was determined as the amount of activity filled in the phantom (measured with an activity meter, which was calibrated for the nuclide and syringe used to fill the phantom with the radioactivity) divided by the volume of the phantom compartment. The means and standard deviations of the RC values of all six spheres were compared.

**Fig. 2:**
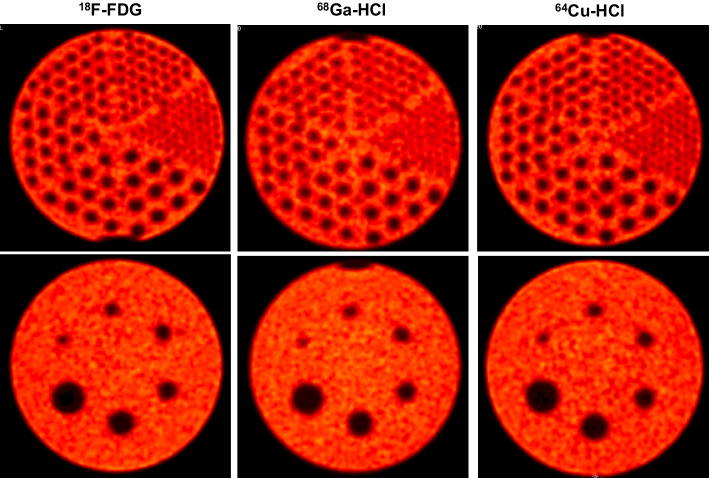
PET images of the Jaszczak phantom filled with ^18^F-FDG (left), ^68^Ga-HCl (middle), or ^64^Cu-HCl (right). Transversal planes at the height of the rods (top) and spheres (bottom). Images were acquired and reconstructed using the same scanner and reconstruction methods and used for qualitative evaluation of spatial resolution

Image noise, called percent background variability in the NEMA NU 2-2018 protocol [14], was calculated as the coefficient of variation within each of the three background VOIs (CoV_BG_). The mean CoV_BG_ values of the three VOIs were compared between PET images of different nuclides.

The contrast between each sphere and the background (contrast recovery coefficient (CRC)) was calculated according to the definitions in the NEMA NU 2-2018 protocol [14]. The means and standard deviations of the CRC of all spheres were compared between PET images.

The contrast–noise ratio (CNR) was calculated as the difference in SUV_mean_ between each sphere and the background divided by the standard deviation of the activity concentration in the background compartment. The means and standard deviations of the CNRs of all spheres were compared between PET images.

According to the NEMA NU 2-2018 protocol [14], the relative count error in the lung insert of the NEMA PET body phantom was determined as the ratio of the average number of counts in a cylindrical VOI with a 30 mm diameter in the lung insert of the phantom not filled with radioactivity relative to the average number of counts within the three VOIs placed in the activity-filled phantom background.

## Results

### Qualitative evaluation of spatial resolution using the Jaszczak phantom

Having adjusted the reconstruction time to the radionuclide-specific half-life, positron yield (Table 1), and activity concentration in the phantom at the timepoint of imaging (Table 2), the total number of true events detected by the PET/CT scanner was similar in the ^18^F-FDG (86.57 Mcts), ^68^Ga-HCl (81.65 Mcts), and ^64^Cu-HCl measurements (85.81 Mcts).

Figure 2 compares the resolution of PET images of the Jaszczak phantom filled with ^18^F-FDG, ^68^Ga-HCl, or ^64^Cu-HCl. In the ^18^F-FDG and ^64^Cu-HCl measurements, the resolvable rods were those separated 4.8 mm apart (rods with the smallest distance in the phantom) as well as all of the more widely spaced rods. In comparison, in the ^68^Ga-HCl measurements, the minimum resolvable rods were those separated 6.4 mm apart (the rods with the second smallest distance in the phantom). The smallest of the six cold spheres featuring a diameter of 9.5 mm was clearly recognizable in PET images of all three nuclides.

**Fig. 3:**
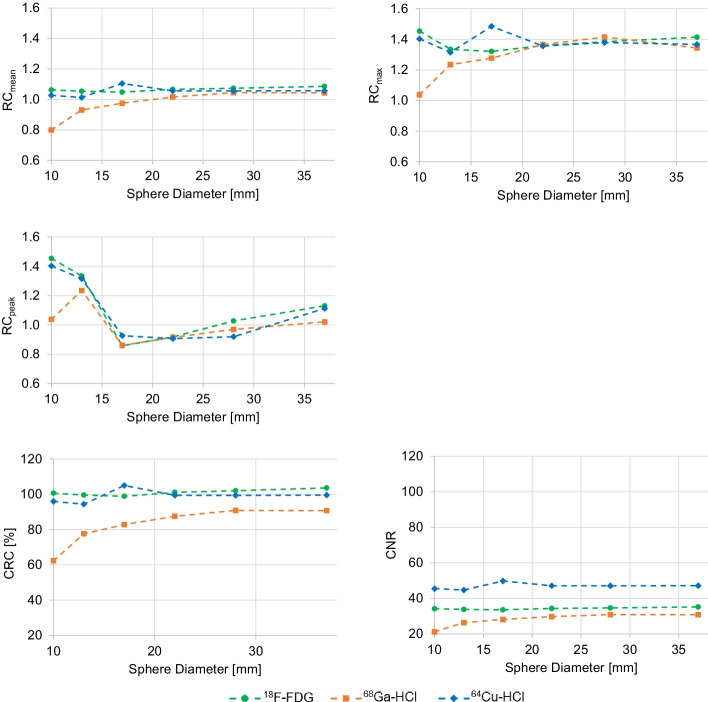
Image quality parameters of the NEMA PET body phantom filled with ^18^F-FDG, ^68^Ga-HCl, or ^64^Cu-HCl. Semiquantitative image quality specified as the mean, maximum and peak recovery coefficient (RC_mean_, RC_max_, and RC_peak_, respectively), percent contrast between each sphere and the background (contrast recovery coefficient (CRC)) and contrast–noise ratio (CNR) for each of the six spheres of the NEMA PET body phantom filled with ^18^F-FDG, ^68^Ga-HCl, or ^64^Cu-HCl at a sphere-to-background activity concentration ratio of approximately 4:1 without the simulation of an obese patient (normal weight setup)

### Analysis of NEMA PET body phantom measurement

With a comparable weight setup of the phantom, the number of true counts detected by the PET/CT scanner was similar for scans of the NEMA PET body phantom filled with ^18^F-FDG, ^68^Ga-HCl, or ^64^Cu-HCl (Table 2). Wrapping the phantom in cooling packs for simulation of attenuation and scatter conditions similar to those in an obese patient reduced the number of true events detected. The decrease in the number of true events detected was similar for all nuclides and ranged between 36.1% for ^68^Ga-HCl measurements at the low contrast ratio and 38.4% for ^68^Ga-HCl measurements at the high contrast ratio (Table 2).

### Semiquantitative evaluation of spatial resolution

When comparing scans of the NEMA PET body phantom at similar sphere-to-background contrast ratios and weight setups, the spatial resolution was similar between ^18^F-FDG and ^64^Cu-HCl PET measurements but worse in the respective ^68^Ga-HCl measurements (Table 3). For each nuclide, spatial resolution was degraded in scans of the phantom mimicking an obese weight setup compared with the respective scan of the phantom not wrapped with cooling packs. The spatial resolution was up to 13% better in the PET image of the phantom filled with a higher versus lower sphere-to-background activity concentration ratio (Table 3: ^18^F-FDG measurements and obese setup).

**Table 3:** Spatial resolution (FWHM) in mm determined using the NEMA PET body phantom

	≈ 4:1 contrast; normal weight setup: mean ± STD (mm)	≈ 4:1 contrast; obese setup: mean ± STD (mm)	≈ 8:1 contrast; normal weight setup: mean ± STD (mm)	≈ 8:1 contrast; obese setup: mean ± STD (mm)
^18^F-FDG	4.55 ± 0.18	4.83 ± 0.32	4.10 ± 0.21	4.21 ± 0.24
^68^Ga-HCl	5.35 ± 0.19	5.35 ± 0.33	4.83 ± 0.16	4.87 ± 0.24
^64^Cu-HCl	4.35 ± 0.20	4.55 ± 0.19	4.17 ± 0.30	4.30 ± 0.20

Spatial resolution is represented by the mean and standard deviation (STD) of the full width at half maximum (FWHM) of all six spheres (in mm) of the NEMA PET body phantom filled with ^18^F-FDG, ^68^Ga-HCl, or ^64^Cu-HCl at sphere-to-background activity concentration ratios of approximately 4:1 or 8:1 and with applied cooling packs around the phantom (obese setup) or without (normal weight setup)

### Semiquantitative evaluation of image quality

Representative of all sphere-to-background activity concentration ratios and weight setups, Fig. 3 compares the RC_mean_, RC_max_, RC_peak_, CRC, and CNR of all spheres of the NEMA PET body phantom filled with ^18^F-FDG, ^68^Ga-HCl, or ^64^Cu-HCl at a sphere-to-background activity concentration ratio of approximately 4:1 and without the simulation of additional attenuation and scattering, as in an obese patient. For all parameters describing image quality, the means and standard deviations of all six spheres are given in Table 4.

**Fig. 4:**
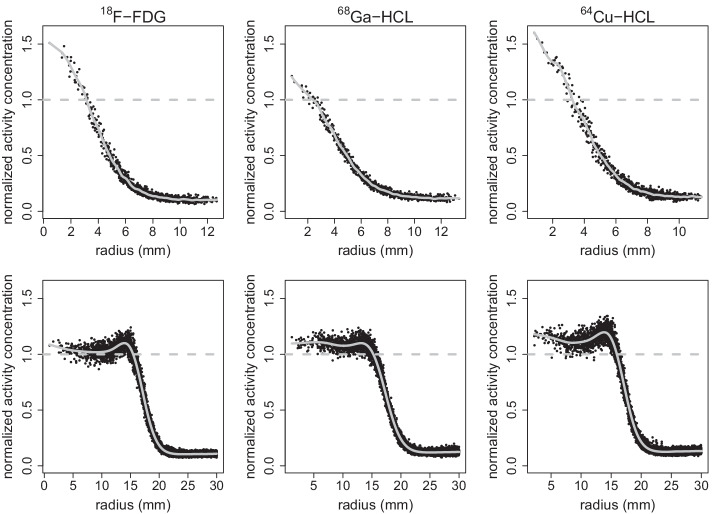
Radial profile of the normalized activity concentration of spheres filled with ^18^F-FDG, ^68^Ga-HCl, or ^64^Cu-HCl. Radial profile of the ratio of measured and true activity concentration of the smallest sphere (upper row; diameter 10 mm) and largest sphere (lower row; diameter 37 mm) of the NEMA PET body phantom filled with ^18^F-FDG, ^68^Ga-HCl, or ^64^Cu-HCl (from left to right) at a sphere-to-background activity concentration ratio of 8:1 without the simulation of an obese patient (normal weight setup). The solid line represents the least-squares fit of the convolution of the point spread function and the object geometry, analyzed to yield the FWHM. To guide the eye, the dashed lines represent a mean ratio of measured and actual activity concentrations of 1

**Table 4:** Means and standard deviations of image quality parameters determined for NEMA PET body phantom measurements

	Contrast: ≈ 4:1: normal weight setup	Contrast: ≈ 4:1: obese setup	Contrast: ≈ 8:1: normal weight setup	Contrast: ≈ 8:1: obese setup
*RC*_*mean*_				
^18^F-FDG	1.06 ± 0.01	1.08 ± 0.02	1.00 ± 0.04	1.02 ± 0.05
^68^Ga-HCl	0.97 ± 0.09	1.03 ± 0.08	0.95 ± 0.06	0.96 ± 0.05
^64^Cu-HCl	1.05 ± 0.03	1.07 ± 0.02	1.10 ± 0.03	1.12 ± 0.03
*RC*_*max*_				
^18^F-FDG	1.38 ± 0.05	1.45 ± 0.04	1.33 ± 0.09	1.37 ± 0.11
^68^Ga-HCl	1.28 ± 0.13	1.41 ± 0.15	1.23 ± 0.04	1.28 ± 0.07
^64^Cu-HCl	1.38 ± 0.06	1.43 ± 0.05	1.42 ± 0.09	1.51 ± 0.06
*RC*_*peak*_				
^18^F-FDG	1.12 ± 0.23	1.10 ± 0.25	1.00 ± 0.33	1.03 ± 0.34
^68^Ga-HCl	1.01 ± 0.13	1.08 ± 0.16	1.00 ± 0.16	0.99 ± 0.21
^64^Cu-HCl	1.10 ± 0.22	1.10 ± 0.27	1.09 ± 0.33	1.13 ± 0.34
*CRC (%)*				
^18^F-FDG	100.99 ± 1.72	101.57 ± 2.99	96.46 ± 4.07	97.86 ± 5.24
^68^Ga-HCl	81.99 ± 10.88	76.48 ± 8.32	80.98 ± 5.75	91.20 ± 5.06
^64^Cu-HCl	98.97 ± 3.67	98.79 ± 2.18	103.90 ± 3.32	106.09 ± 3.09
*CNR*				
^18^F-FDG	34.26 ± 0.58	24.60 ± 0.72	86.55 ± 3.65	64.19 ± 3.44
^68^Ga-HCl	27.83 ± 3.69	19.36 ± 2.11	70.48 ± 5.00	61.10 ± 3.39
^64^Cu-HCl	46.86 ± 1.74	33.24 ± 0.73	76.40 ± 2.44	57.87 ± 1.69
*CoV*_*BG*_* (%)*				
^18^F-FDG	8.96 ± 0.33	12.55 ± 0.15	9.72 ± 0.21	13.29 ± 0.77
^68^Ga-HCl	9.60 ± 0.40	12.87 ± 0.41	9.67 ± 0.43	12.56 ± 0.81
^64^Cu-HCl	9.30 ± 0.64	13.09 ± 0.63	9.68 ± 0.69	13.04 ± 0.77
*Lung count error [%]*				
^18^F-FDG	3.82	5.42	4.19	5.22
^68^Ga-HCl	7.85	13.48	8.23	5.90
^64^Cu-HCl	3.72	5.11	3.76	4.95

Means and standard deviations of the mean, maximum and peak recovery coefficients (RC_mean_, RC_max_, and RC_peak_, respectively), percent contrast between each sphere and the background (contrast recovery coefficient (CRC)), contrast–noise ratio (CNR), percent background variability (CoV_BG_) and the relative count error in the lung insert (lung count error). Except for the relative count error in the lung insert, each parameter is presented as the mean ± standard deviation of the six spheres for PET/CT measurements of the NEMA PET body phantom filled with ^18^F-FDG, ^68^Ga-HCl, or ^64^Cu-HCl with sphere-to-background activity concentration ratios of approximately 4:1 or 8:1 and with (obese setup) or without (normal weight setup) the simulation of an obese patient by applying cooling packs around the phantom

The mean activity concentrations recovered from the PET/CT images were comparable with the true activity concentrations for all activity-filled spheres of different diameters in ^18^F-FDG measurements of the NEMA PET body phantom, as indicated by RC_mean_ ≈ 1 for all spheres (Fig. 3). These findings were independent of the contrast ratio or weight setup (Table 4). Similarly, the RC_mean_ values were comparable between spheres of different diameters in all ^64^Cu-HCl measurements. In comparison, RC_mean_ differed between spheres of different sizes in the ^68^Ga-HCl PET/CT scans. Spheres with a diameter of  10 mm showed the largest differences compared with the true activity concentration within the sphere, while recovery improved with increasing sphere size, and RC_mean_ reached ≈ 1 for the largest spheres (Fig. 3). For example, in ^68^Ga-HCl PET/CT scans of the phantom filled with a sphere-to-background activity concentration ratio of approximately 8:1 and with normal weight setup, RC_mean_ differed between 0.87 and 1.02 for the sphere of the smallest and largest diameter.

While the measurements of the NEMA PET body phantom filled with ^64^Cu-HCl at low sphere-to-background activity concentration ratios revealed an RC_mean_ of approximately 1 for all spheres, the mean activity concentration recovered from all spheres was on average 10 to 12% higher than the assumed true activity concentration in the ^64^Cu-HCl measurements at higher sphere-to-background activity concentration ratios (Table 4).

The maximal recovered activity concentrations were much higher than the true activity concentrations in PET/CT measurements of all nuclides, with the lowest RC_max_ values in ^68^Ga-HCl measurements and the highest RC_max_ in ^64^Cu-HCl measurements (Table 4). In the ^64^Cu-HCl and ^18^F-FDG but not ^68^Ga-HCl measurements, the smallest sphere of 10 mm diameter featured higher RC_max_ values than the remaining spheres.

The mean CRC values of spheres of different diameters were similar in the ^64^Cu-HCl and ^18^F-FDG measurements but lower in the ^68^Ga-HCl measurements (Table 4). For each nuclide, the mean CRC values were comparable between measurements of the phantom with different weight setups and sphere-to-background activity concentration ratios. Figure 3 illustrates that the CRC was independent of the size of the sphere in ^64^Cu-HCl and ^18^F-FDG measurements but decreased continuously with decreasing sphere diameter in ^68^Ga-HCl measurements. In all ^64^Cu-HCl and ^18^F-FDG measurements, CNR was comparable between spheres of different sizes. In comparison, in ^68^Ga-HCl measurements, CNR decreased with decreasing sphere diameter (Fig. 3). For example, in ^68^Ga-HCl measurements with a normal weight setup and a high contrast ratio, the CNR of the smallest sphere was 33% lower than that of the largest sphere. CoV_BG_ was similar for all nuclides when comparing PET images of the NEMA PET body phantom with the same setup (weight setup and sphere-to-background activity concentration ratio). CoV_BG_ was lower in the normal weight setup than in the specific obese setup.

For measurements with all phantom setups, the relative count error in the lung insert was higher for ^68^Ga-HCl measurements than for ^18^F-FDG or ^64^Cu-HCl measurements (Table 4).

## Discussion

The main finding of this study is that the image quality of PET scans of a clinical, digital PET/CT scanner (especially spatial resolution, recovery coefficients, and image noise) is strongly affected by the positron energy of the PET isotope used and by attenuation.

### Qualitative and semiquantitative evaluation of spatial resolution

Both the qualitative analyses of the Jaszczak phantom and the semiquantitative analyses of the NEMA PET body phantom revealed that spatial resolution was degraded in ^68^Ga-HCl versus ^18^F-FDG and ^64^Cu-HCl PET measurements. Similarly, studies in preclinical [10] and clinical PET scanners [20, 21] have found worse spatial resolution in ^68^Ga measurements than in ^18^F PET measurements. In contrast to the study by Sanchez-Crespo et al., who used similar activity concentrations and acquisition times for ^18^F and ^68^G PET measurements, in this study the count rates between PET scans with the different nuclides were kept the same. The fact that differences are still visible implies that the higher endpoint energy of positrons originating from the decay of ^68^Ga (and an associated increase in positron range) compared with those originating from the decay of ^18^F or ^68^Ga worsens spatial resolution in clinical, digital PET/CT scans. The image resolution was similar between ^64^Cu-HCl and ^18^F-FDG PET/CT measurements, as the positrons arising from the decay of those two nuclides feature similar endpoint energies.

Consistent with Rogasch et al. [22], we found that the spatial resolution of PET data that were reconstructed using iterative reconstruction and PSF degrades when the signal-to-background ratios decrease. This is most likely a combined effect of the dependence of the spatial resolution on the optimized number of iterations and subsets during iterative PET reconstruction and a dependency of the magnitude of the Gibbs artifacts during PSF reconstruction on the signal-to-background ratio [22]. Similarly, degraded spatial resolution in scans of the NEMA PET body phantom mimicking an obese weight setup is most likely a result of the iterative reconstruction of the PET data and a nonoptimized number of iterations and subsets. In this study, PET data were reconstructed according to the standards in our clinical routine for ^18^F-FDG to best replicate clinical practice. This implies an iterative reconstruction of the PET data and a fixed number of iterations and subsets. An optimization of the reconstruction parameters is beyond the scope of this analysis.

### Semiquantitative evaluation of image quality

The similar number of true events detected by the PET/CT scanner in the measurements of the NEMA PET body phantom filled with the different nuclides at the normal or obese weight setup (Table 2) indicates that the adjustment of the analyzed recording time accurately compensated for nuclide-specific differences in positron yield and half-life. There were small deviations between targeted and actual activity concentrations or contrast ratios in the phantom at the timepoint of the PET/CT scans. However, this did not affect the analyses, as the activity concentrations recovered from the PET/CT images were normalized to actual activity concentrations in the phantom and as the statistics were kept comparable between the scans of the phantom at similar weight setups.

In all ^18^F-FDG and ^64^Cu-HCl measurements, the mean activity concentrations recovered from the PET/CT images were comparable between spheres of different sizes. In comparison, in the ^68^Ga-HCl measurements, the RC_mean_ values were lower for spheres of ≤ 13 mm diameter but reached ≈ 1 for spheres ≥ 22 mm diameter. The lower RC values for the smallest spheres filled with ^68^Ga-HCl are most likely a result of the degraded spatial resolution of smaller objects in ^68^Ga-PET/CT scans. In agreement with the presented ^68^Ga-HCl measurements but differing from the ^18^F-FDG measurements, both Sanchez-Crespo et al. [20] and Ryu et al. [21] found that the scanned object should be at least 20 mm in diameter to accurately recover the true mean activity concentration in ^18^F-FDG and ^68^Ga-HCl images of a clinical PET/CT scanner. The different findings in the ^18^F-FDG measurements presented in this study are most likely due to the use of the latest PET/CT scanning technology. In this study, a ToF-enabled PET/CT scanner was used, which has been shown to exhibit less size-based partial volume bias than non-ToF scanners [23].

While the mean activity concentrations were accurately recovered in all ^18^F-FDG measurements and in ^64^Cu-HCl measurements at low sphere-to-background activity concentration ratios (RC_mean_ ~ 1), the recovered mean activity concentrations were approximately 10% higher than the true activity concentration in all spheres when the phantom was filled with ^64^Cu-HCl at sphere-to-background activity concentration ratios of approximately 8:1. As RC_mean_ was increased equally for all spheres of this measurement series, we assume that inaccuracies have occurred in determining the true activity concentration. Measurement inaccuracies can occur when measuring low radioactivity values with the gamma counter. These inaccuracies are larger for ^64^Cu-HCl than for ^18^F-FDG or ^68^Ga-HCl due to the much lower β^+^ yield (Table 1). In addition, the determination of the volume of solution in which the radioactivity was diluted may have been incorrect. This may have resulted in an overestimation of the activity concentration. However, small underestimations of the true activity concentration are not decisive for the core message of this study: the consistency of the image quality parameters for spheres of different diameters.

The PSF reconstruction used in this study leads not only to an improved sharpness of hot spheres and higher spatial resolution [25] but also to an artificial edge overemphasis, which is called the Gibbs artifact [24, 26]. In addition, ToF and the use of an inappropriate kernel can cause edge artifacts and affect contrast recovery and spatial resolution [27]. The artificial increase in the retrieved activity concentration, which is particularly pronounced at the edge of a lesion, can be seen in the radial profiles shown in Fig. 4. The overestimation of the retrieved activity concentration is nonlinear and increases with decreasing lesion size (Fig. 4) [24]. This explains the higher RC_max_ values of the smallest sphere of 10 mm diameter compared with larger spheres, as found in our ^18^F-FDG and ^64^Cu-HCl measurements.

The magnitude of the Gibbs artifacts might have been comparable between the measurements with the different nuclides as the same reconstruction parameters were used. However, partial volume effects were presumably higher in the ^68^Ga-HCl measurements than in the ^18^F-FDG or ^64^Cu-HCl measurements as the mean range of positrons in water originating from the decay of ^68^Ga is higher (Table 1). This may have outweighed the nonlinear overestimation of the retrieved activity concentration, which in combination resulted in lower RC_mean_ and RC_max_ values in the ^68^Ga-HCl measurements than in the ^18^F-FDG or ^64^Cu-HCl measurements, especially for the smallest sphere.

The higher positron range and partial volume effects in PET measurements with ^68^Ga potentially reduce CRC and CNR in ^68^Ga-HCl measurements compared with ^64^Cu-HCl or ^18^F-FDG measurements. This is particularly relevant for small lesions and might explain the decrease in CNR and CRC with decreasing sphere diameter. Similarly, Ryu et al. reported lower CRC values in ^68^Ga-PET scans than in ^18^F PET scans for spheres of 10 mm to 37 mm diameter [21]. While Ryu et al. reported a decrease in CRC with decreasing sphere size in ^68^Ga-HCl and ^18^F-FDG measurements, the CRC values in our ^18^F-FDG measurements were comparable between all spheres and approximately 100%. The particularly low CRC values for spheres ≤ 13 mm in diameter filled with ^18^F-FDG in [21] are potentially due to a reconstruction of the data without resolution recovery by PSF or worse ToF performance [21].

The higher relative count error in the lung insert of the NEMA PET body phantom filled with ^68^Ga-HCl compared to ^18^F-FDG or ^64^Cu-HCl was probably caused by a combination of factors. The range of the positrons originating from the decay of ^68^Ga is higher, which results in a greater penetration of the lung insert. In addition, single-photon emission and scattered photons arise during the decay of ^68^Ga but not during the decay of ^18^F or ^64^Cu.

Wrapping the phantom in cooling packs increased the scattering and absorption of photons and thus reduced the detected number of true counts, which increased CoV_BG_ to a similar extent in PET measurements with all nuclides.

## Conclusion

In summary, the image quality was found to be similar between ^18^F-FDG and ^64^Cu-HCl PET/CT images featuring similar maximal endpoint energies of positions and thus a similar maximum range of positrons in tissue before annihilation. In comparison, the much higher endpoint energy of positrons arising from the decay of ^68^Ga degrades quantitative parameters describing image quality (RC, CRC, and CNR) and spatial resolution, especially of small lesions ≤ 13 mm in diameter. As a result, in ^68^Ga-HCl images but not in ^18^F-FDG and ^64^Cu-HCl PET/CT images, quantitative image parameters differ between lesions of different sizes.

## Supplementary Information

**Additional file 1: **Transversal (left), coronal (middle), and sagittal plane of the NEMA PET body phantom filled with 18F-FDG and illustration of the regions of interest.

## Data Availability

The datasets used and/or analyzed during the current study are available from the corresponding author on reasonable request.

## Abbreviations

SUV: Standardized uptake value
ToF: Time of flight
PSF: Point spread function
HCl: Hydrochloric acid
FoV: Field of view
OSEM: Ordered subset expectation maximization
FWHM: Full width at half maximum
VOI: Volume of interest
RC: Recovery coefficient
CoV_BG_: Coefficient of variation within the background VOI
CRC: Contrast recovery coefficient
CNR: Contrast–noise ratio
