# Supplementary material for: Comparison of image quality and spatial resolution between 18F, 68Ga, and 64Cu phantom measurements using a digital Biograph Vision PET/CT
Source: EJNMMI Phys. 2022 Sep 5;9:58. doi: 10.1186/s40658-022-00487-7 (PMC9445107; doi:10.1186/s40658-022-00487-7)
Supplement: Supplementary file 1 — Additional file 1: Transversal (left), coronal (middle), and sagittal plane of the NEMA PET body phantom filled with 18F-FDG and illustration of the regions of interest. [file 40658_2022_487_MOESM1_ESM.docx]

**Comparison of image quality and spatial resolution between ^18^F, ^68^Ga and ^64^Cu phantom measurements using a digital Biograph Vision PET/CT**

Authors

Anja Braune; University Hospital Carl Gustav Carus at the Technische Universität Dresden, Department of Nuclear Medicine, Dresden, Germany;
anja.braune@uniklinikum-dresden.de (corresponding author)

Liane Oehme; University Hospital Carl Gustav Carus at the Technische Universität Dresden, Department of Nuclear Medicine, Dresden, Germany;
[liane.oehme@uniklinikum-dresden.de](mailto:liane.oehme@uniklinikum-dresden.de)

Robert Freudenberg; University Hospital Carl Gustav Carus at the Technische Universität Dresden, Department of Nuclear Medicine, Dresden, Germany;
[robert.freudenberg@uniklinikum-dresden.de](mailto:robert.freudenberg@uniklinikum-dresden.de)

Frank Hofheinz; PET Center, Institute of Radiopharmaceutical Cancer Research, Helmholtz-Zentrum Dresden-Rossendorf, Dresden, Germany; [hofheinz@hzdr.de](mailto:hofheinz@hzdr.de)

Jörg van den Hoff; PET Center, Institute of Radiopharmaceutical Cancer Research, Helmholtz-Zentrum Dresden-Rossendorf, Dresden, Germany; [j.van_den_hoff@hzdr.de](mailto:j.van_den_hoff@hzdr.de)

Jörg Kotzerke; University Hospital Carl Gustav Carus at the Technische Universität Dresden, Medizinische Fakultat Carl Gustav Carus, Department of Nuclear Medicine, Dresden, Germany;
[joerg.kotzerke@uniklinikum-dresden.de](mailto:joerg.kotzerke@ukdd.de)

Sebastian Hoberück; University Hospital Carl Gustav Carus at the Technische Universität Dresden, Department of Nuclear Medicine, Dresden, Germany;
Helios Klinikum Erfurt, Erfurt, Germany.
[Sebastian.Hoberueck@uniklinikum-dresden.de](mailto:Sebastian.Hoberueck@uniklinikum-dresden.de)

Keywords: PET/CT, ^18^F-FDG, ^68^Ga, ^64^Cu, NEMA PET Body phantom, Jaszczak phantom, spatial resolution, image quality

Journal: EJNMMI Physics

**Attachment – Figure 1A:** Transversal (left), coronal (middle), and sagittal plane of the NEMA PET Body phantom filled with ^18^F-FDG and illustration of the regions of interest.


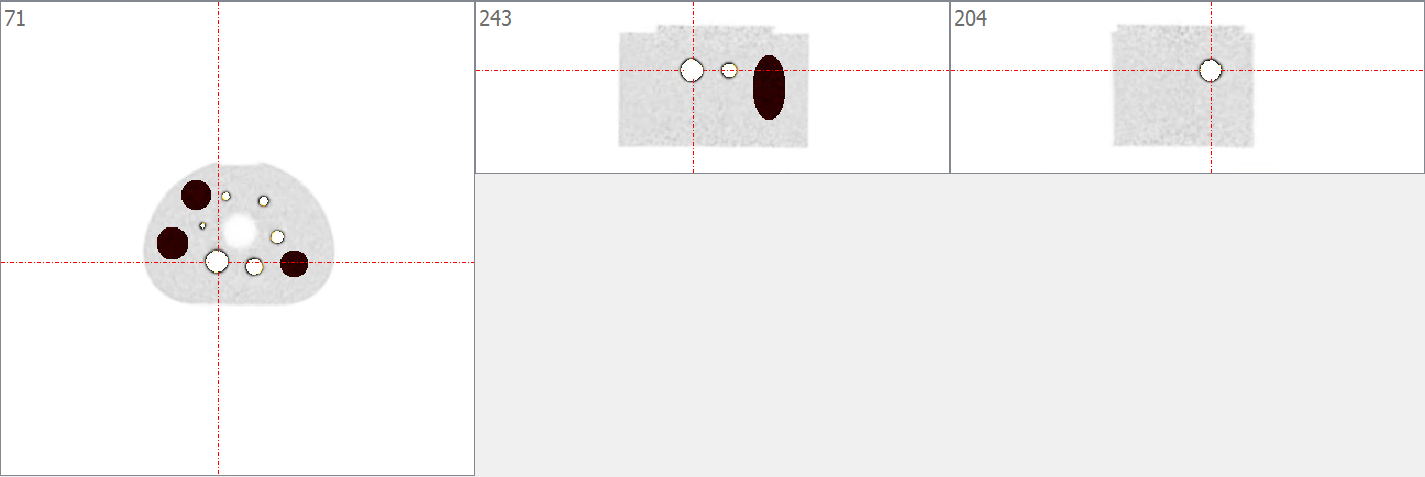


**Figure Legend:** Regions of interest of the spheres are shown in white and the three uniform background regions are shown in black. Regions of interest were used for semi-quantitative evaluation of image quality.
